# Ultrasound Features of Uterine Perivascular Epithelioid Cell Tumor (PEComa): A Systematic Review

**DOI:** 10.3390/jimaging12060268

**Published:** 2026-06-18

**Authors:** Laura Grazia Zompì, Giorgio Maria Baldini, Maria Bardi, Salvatore Lopez, Angela Calabrese, Maria Antonietta Ramunno, Giuseppe Colonna, Vera Loizzi, Francesca Arezzo, Gennaro Cormio

**Affiliations:** 1 Department of Interdisciplinary Medicine (DIM), University of Bari Aldo Moro, 70124 Bari, Italy; m.bardi994@gmail.com (M.B.); gennaro.cormio@uniba.it (G.C.); 2 Gynecologic Oncology Unit, IRCCS Istituto Tumori Giovanni Paolo II, 70124 Bari, Italy; s.lopez@oncologico.bari.it (S.L.); giuseppe.colonna@uniba.it (G.C.); vera.loizzi@uniba.it (V.L.); francescaromanaarezzo@gmail.com (F.A.); 3 Department of Radiology, IRCCS Istituto Tumori Giovanni Paolo II, 70124 Bari, Italy; a.calabrese@oncologico.bari.it; 4 Department of Pathology, IRCCS Istituto Tumori Giovanni Paolo II, 70124 Bari, Italy; ramunno.m.ant@gmail.com; 5 Department of Translational Biomedicine and Neurosciences (DiBraiN), University of Bari Aldo Moro, 70124 Bari, Italy

**Keywords:** uterine PEComa, perivascular epithelioid cell tumor, transvaginal ultrasound, systematic review, leiomyosarcoma, differential diagnosis, MUSA

## Abstract

Uterine perivascular epithelioid cell tumor (PEComa) is a rare mesenchymal neoplasm whose sonographic profile has not been systematically characterized. We describe an index case of malignant uterine PEComa and present a PRISMA 2020-compliant systematic review (PubMed, Scopus, Cochrane Library; search 1 March 2026) of studies reporting original ultrasound data of histologically confirmed uterine PEComa. Sonographic features were coded with MUSA/IETA terminology; Clopper–Pearson 95% confidence intervals (CI) were calculated for key proportions, and malignancy subgroups were summarized descriptively. Thirty-one cases were pooled (30 from 18 studies plus our index case; median age 41 years). The profile comprised absent acoustic shadowing in all documented cases (10/10; 95% CI 69.2–100%), moderate-to-abundant vascularisation (Color Score 3–4, 91.7%), variable echogenicity (heterogeneous 56.0%) and predominantly regular margins (69.6%). Preoperative misdiagnosis occurred in 100% of cases, most often as leiomyoma (41.4%). In cases with known malignancy status (n = 17), irregular margins and cystic areas appeared more often in malignant lesions, but subgroups were too small for testing. Only 4/18 studies applied standardized terminology. Uterine PEComa shows a recurrent pattern of absent shadowing, high vascularisation and solid consistency with regular margins that may aid differential diagnosis; systematic adoption of MUSA/IETA terminology in future reports is strongly advocated.

## 1. Introduction

Perivascular epithelioid cell tumors (PEComas) are rare mesenchymal neoplasms defined by a characteristic myomelanocytic immunophenotype, with co-expression of smooth muscle markers (smooth muscle actin, desmin) and melanocytic markers (HMB-45, Melan-A, MiTF) [1,2].

Originally described in the 1990s as a family of tumors sharing this distinctive immunoprofile, PEComas may arise virtually anywhere in the body, including the kidney, lung, retroperitoneum, liver, and the female genital tract [1]. Within the genital tract, the uterine corpus is the most common site of origin, accounting for the majority of reported cases both in women with and without tuberous sclerosis complex (TSC) [1].

Approximately 15–20% of uterine PEComas are associated with TSC, a genetic syndrome caused by germline or somatic mutations in the TSC1 or TSC2 tumor suppressor genes, which leads to constitutive activation of the mTOR (mammalian target of rapamycin) signaling pathway [1,2,3]; in the largest single-institution series to date, Bennett et al. reported a TSC association in 13% of classic uterine PEComas [1]. This mechanism supports the use of mTOR inhibitors (e.g., sirolimus, temsirolimus, nab-sirolimus) in advanced disease [4,5]. Histopathological stratification into benign, malignant, and uncertain malignant potential categories, as proposed by Folpe et al. [6], is based on the presence of ≥2 of the following features: tumor size > 5 cm, high nuclear grade, mitotic rate > 1/50 high-power fields (HPF), necrosis, vascular invasion, and infiltrative growth pattern.

Uterine PEComa most commonly presents in women in the fourth to sixth decade of life with abnormal uterine bleeding (AUB), pelvic pain, or as an incidental finding, symptoms that overlap substantially with those of far more prevalent uterine pathologies including leiomyoma, leiomyosarcoma, and endometrial polyps [1,3]. Transvaginal ultrasound (US) is the established first-line imaging modality in the evaluation of any uterine mass. However, the sonographic profile of uterine PEComa has not been systematically described. Available imaging data are fragmented across isolated case reports and small series [7], none of which has consistently applied standardized ultrasound terminology such as the Morphological Uterus Sonographic Assessment (MUSA) [8] or International Endometrial Tumor Analysis (IETA) [9] consensus frameworks.

In this study we first describe the ultrasound features of an index case of malignant uterine PEComa treated at our institution. We then report a PRISMA 2020-compliant systematic review [10] of the published ultrasound literature on uterine PEComa. The aim was to define a reproducible sonographic profile that may improve preoperative recognition of this rare tumor. We pooled and described the gray-scale and Doppler features using MUSA and IETA terms, measured how often these tumors were misdiagnosed before surgery and which lesions they were mistaken for, and descriptively evaluated whether any feature differed by malignancy category.

## 2. Case Report

A 60-year-old woman (gravida 5, para 2) was referred to our institution for a second gynecological opinion after an isolated episode of post-menopausal metrorrhagia.

The patient was a previously healthy woman with no relevant past medical or surgical history. She was not taking any medications, and all prior routine gynecological evaluations had been unremarkable. One month before presentation, she experienced an isolated episode of metrorrhagia and underwent endometrial curettage. Histopathological examination of the endometrial tissue showed no evidence of malignancy.

**Figure 1:**
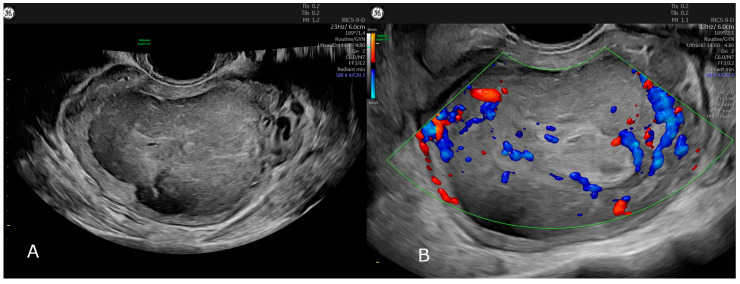
(**A**) Grayscale transvaginal ultrasound of the index case. A 53 × 39 × 58 mm heterogeneous myometrial mass is identified at the uterine fundus, classified FIGO type 2–5. The lesion displays irregular multilobulated margins, mixed isoechoic and hyperechoic internal texture, extension to the fundal serosa (residual myometrial margin < 1 mm), and complete absence of posterior acoustic shadowing. No calcifications are visible. (**B**) Color Doppler transvaginal ultrasound of the same lesion. A Color Score (CS) of 3 is documented, reflecting moderate intralesional vascularization.

On gynecological examination, the uterine body appeared enlarged in both size and consistency. Additionally, the patient reported pelvic fullness in the suprapubic region and abdominal bloating. Transvaginal ultrasound revealed a myometrial lesion located at the uterine fundus, classified as FIGO type 2–5 (i.e., a transmural “hybrid” lesion with both submucosal and subserosal components, with ≥50% of the mean diameter located within the myometrium on each side), with irregular, multilobulated margins, measuring 53 × 39 × 58 mm. It showed heterogeneous echogenicity, mostly isoechoic with hyperechoic areas. The lesion extended to the fundal serosa, with a residual myometrial free margin of less than 1 mm. Absence of acoustic shadowing was noted. Color Doppler evaluation of the mass revealed a Color Score of three according to the IOTA (International Ovarian Tumor Analysis) criteria (Figure 1), reflecting moderate intralesional vascularisation. Adnexa appeared normal. There was no free fluid in the pouch of Douglas.

**Figure 2:**
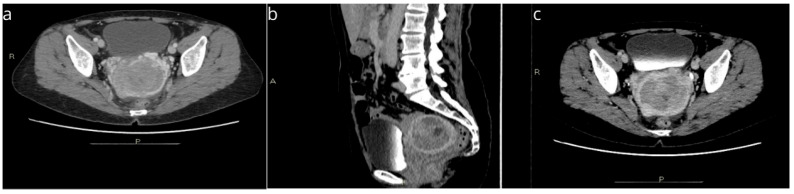
(**a**) Sagittal CT image demonstrating an enlarged, heterogeneous uterus. A large solid mass with irregular internal attenuation occupies the uterine corpus. No involvement of adjacent pelvic structures is identified. (**b**) Axial CT image at the level of the uterine body showing the maximum cross-sectional extent of the mass (approximately 76 × 68 mm) with avid peripheral enhancement and central hypodense necrotic areas. The uterine contour is preserved and no evidence of serosal infiltration of adjacent structures is observed. (**c**) Portal-phase axial CT image confirming absence of peritoneal carcinomatosis, pathological lymphadenopathy, or distant metastatic spread.

CT staging was performed to characterize the pelvic mass further and to exclude distant metastatic disease. CT demonstrated an enlarged, heterogeneous uterus containing a dominant solid hypervascular mass with maximum axial dimensions of 76 × 68 mm (Figure 2). The larger CT measurement relative to ultrasound likely reflects the inclusion of adjacent satellite nodules (ranging from 5 to 15 mm in diameter, identified at macroscopic examination) within the CT measurement volume, as well as differences in measurement plane. No peritoneal implants, lymphadenopathy, or distant metastases were identified.

On the basis of lesion size, marked vascularisation, and rapid clinical onset, a uterine tumor of uncertain malignant potential was suspected.

Total abdominal hysterectomy with bilateral salpingo-oophorectomy (BSO) was performed via laparotomy, with additional peritoneal washings and omental biopsies.

Macroscopic examination of the surgical specimen revealed a solid yellowish nodular neoplasm within the uterine body measuring 60 mm in maximum diameter, with a central area of necrotic appearance and multiple contiguous satellite nodules ranging from 5 to 15 mm.

Microscopically, the neoplasm exhibited high cellularity and was composed predominantly of epithelioid cells, with a minor component of spindle-shaped elements. The tumor cells had eosinophilic to clear cytoplasm, marked pleomorphism, severe cytologic and nuclear atypia, and a high mitotic rate (32 mitoses per 50 HPF), including the presence of atypical mitotic figures. Tumor necrosis and evidence of endovascular tumor infiltration were observed. Multiple peritumoral nodules with identical morphological features were also present.

**Figure 3:**
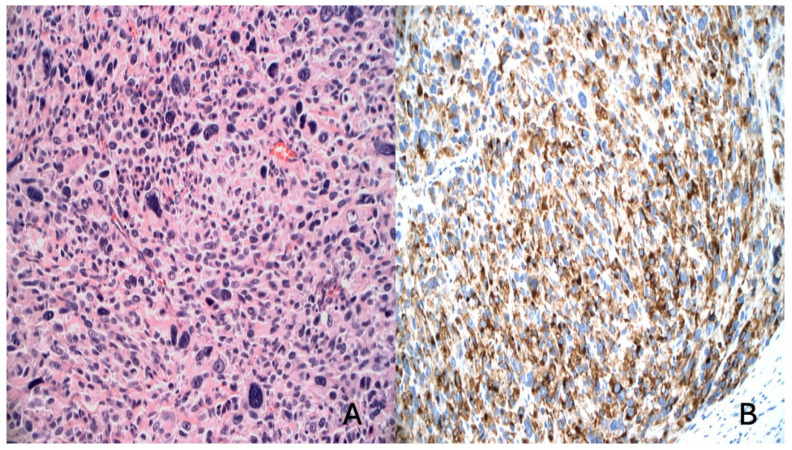
(**A**) Haematoxylin and Eosin (H&E)-stained section. The characteristic perivascular growth pattern is evident, with epithelioid tumor cells arranged concentrically around thin-walled vascular channels. (**B**) Immunohistochemical staining for HMB-45. The neoplastic epithelioid cells demonstrate diffuse, strong granular cytoplasmic positivity, confirming the melanocytic component of the myomelanocytic immunophenotype. This co-expression of a melanocytic marker in a smooth-muscle-forming tumor is characteristic of the PEComa family of tumors.

Immunohistochemically, the neoplastic cells showed diffuse positivity for smooth muscle actin (SMA) and HMB-45, and were positive for estrogen receptor (ER). The tumor was negative for Melan-A, SOX-10, S100, progesterone receptor (PgR), and cyclin D1 (Figure 3).

The combined morphological and immunophenotypic profile was diagnostic of malignant PEComa, satisfying four Folpe criteria: tumor size > 5 cm, high nuclear grade, mitotic rate > 1/50 HPF, and tumor necrosis [6].

## 3. Materials and Methods

### 3.1. Study Design and Registration

This systematic review was conducted and reported in accordance with the 2020 Preferred Reporting Items for Systematic Reviews and Meta-Analyses (PRISMA) guidelines [10].

### 3.2. Eligibility Criteria

Case report and case series reporting original clinical and sonographic data of patients diagnosed with uterine PEComa were included. 

Studies were excluded if they lacked an ultrasonographic description of the lesion, focused only on the molecular or genetic aspects of PEComa, were not available in full text or not in English, or were review articles without an original case.

### 3.3. Information Sources and Search Strategy

A comprehensive electronic search was conducted on 1 March 2026 across three databases: PubMed/MEDLINE, Scopus, and the Cochrane Library. The following search string was applied to PubMed/MEDLINE: (PEComa[Title/Abstract] OR “Perivascular Epithelioid Cell Tumor”[Title/Abstract] OR “Perivascular Epithelioid Cell Neoplasm”[Title/Abstract]) AND (uterus[Title/Abstract] OR uterine[Title/Abstract] OR “Uterine Neoplasms”[MeSH Terms]) AND (ultrasound[Title/Abstract] OR ultrasonography[Title/Abstract] OR sonography[Title/Abstract] OR “Ultrasonography”[MeSH Terms] OR Doppler[Title/Abstract]). The following equivalent free-text string was adapted for Scopus: (TITLE-ABS-KEY(PEComa OR “Perivascular Epithelioid Cell Tumor” OR “Perivascular Epithelioid Cell Neoplasm”)) AND (TITLE-ABS-KEY(uterus OR uterine)) AND (TITLE-ABS-KEY(ultrasound OR ultrasonography OR sonography OR Doppler)). For the Cochrane Library, the following MeSH-free string was used in title, abstract, and keyword fields: (PEComa OR perivascular epithelioid) AND (uterus OR uterine) AND (ultrasound OR ultrasonography OR Doppler). No date or publication-type restrictions were applied at the search stage. English-language restriction was applied at the eligibility stage (Section 3.2). The search was limited to studies involving human subjects. Three complementary databases (PubMed/MEDLINE, Scopus and the Cochrane Library) were selected to balance biomedical coverage with feasibility; Scopus provides broad indexing that substantially overlaps with Embase and Web of Science for the biomedical case-report literature relevant to this rare tumor. Embase, Web of Science and IEEE Xplore were not searched separately: Embase was not available through our institutional license, while Web of Science and IEEE Xplore were judged unlikely to add uterine-PEComa ultrasound cases beyond those captured by PubMed and Scopus, given the clinical nature of the literature. Gray literature and conference abstracts were not systematically searched. These choices are acknowledged as a limitation in Section 5.

### 3.4. Study Selection

This systematic review was not prospectively registered in PROSPERO or any other protocol registry. The review protocol, including predefined eligibility criteria, search strategy, and data extraction form, was developed a priori and is publicly available at https://doi.org/10.17605/OSF.IO/EUQBF.

Retrieved records were deduplicated and imported into a reference management tool. Title and abstract screening was performed independently by two reviewers (G.M.B. and L.G.Z.); inter-reviewer agreement was quantified using Cohen’s kappa. Agreement was complete at both the title/abstract and full-text stages (κ = 1.00), so no disagreements required consensus discussion or third-party arbitration. This complete agreement reflects the narrow and unambiguous eligibility criteria, which made the large majority of exclusion decisions clear-cut at the title and abstract level. The individual screening logs of both reviewers and the agreement calculation are publicly available at the DOI cited in Section 3.1.

**Figure 4:**
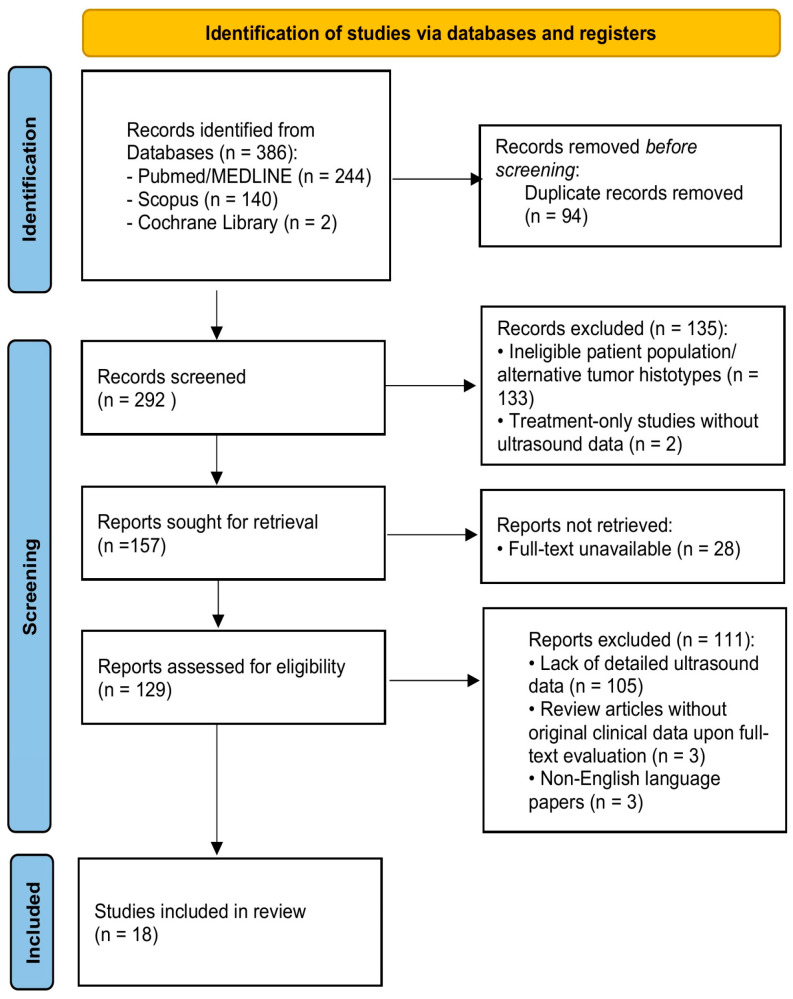
PRISMA 2020 [10] flow diagram of the literature search and study selection. Three databases were searched on 1 March 2026: PubMed/MEDLINE (n = 244), Scopus (n = 140), and the Cochrane Library (n = 2), yielding 386 records. After removal of 94 duplicates, 292 records were screened; 135 were excluded at title and abstract (133 not describing uterine PEComa and 2 treatment-only studies without ultrasound data). Of 157 reports sought, 28 were not retrieved, and 129 were assessed for eligibility; 111 were excluded at full text (105 lacking a detailed ultrasound description, 3 reviews without an original case, 3 non-English publications), leaving 18 included studies. The institutional index case is described in the text and is not part of the database search.

Full-text assessment was subsequently performed by both reviewers, applying the pre-specified inclusion and exclusion criteria. The full selection process is documented in the PRISMA 2020 [10] flow diagram (Figure 4).

### 3.5. Data Extraction

Data extraction was performed independently by two reviewers (G.M.B. and L.G.Z.) using a standardized extraction form developed a priori. The following variables were collected for each included case: study characteristics (first author, year, study design, number of cases); patient demographics (age, clinical presentation); lesion characteristics (maximum diameter, location, FIGO classification when reported); sonographic features (echogenicity, margins, presence of cystic areas, acoustic shadowing, vascular pattern, Color Score); application of MUSA [8] or IETA [9] standardized terminology; MRI findings when available; preoperative diagnosis; histopathological malignancy classification; and Folpe criteria satisfied when reported.

### 3.6. Quality Assessment

The methodological quality and risk of bias of each included study were assessed independently by two reviewers (G.M.B. and L.G.Z.) using the Joanna Briggs Institute (JBI) Critical Appraisal Checklist for Case Reports, comprising eight binary items (Yes/No/Unclear): Q1 (demographic characteristics), Q2 (patient history), Q3 (current clinical condition), Q4 (diagnostic tests and results), Q5 (intervention and outcomes), Q6 (post-intervention condition), Q7 (adverse events), and Q8 (take-away lessons). Each item was scored as Yes = 1 or No/Unclear = 0; the total score ranged from 0 to 8. No study was excluded on the basis of quality score alone; quality assessment findings were used to contextualize the evidence.

### 3.7. Statistical Analysis

Continuous variables (age, lesion diameter) were summarized as median and interquartile range (IQR, 25th–75th percentile) with full range, computed on cases with available data; the mean is reported for reference only. Non-parametric summaries were chosen a priori in view of the small number of evaluable cases. Categorical variables were reported as absolute frequencies and percentages (n/N, %); the denominator N represents the number of cases for which the specific parameter was documented (i.e., not reported as NR), and is stated explicitly for each variable. Missing data were handled by complete-case analysis on a per-parameter basis: no imputation was performed, and parameters reported as NR were excluded from the denominator of the corresponding variable rather than being counted as feature-absent. Because the proportion of unreported parameters was high and likely non-random (informative missingness), the robustness of the two most affected estimates, namely absence of acoustic shadowing and Color Score 3–4, was explored with a best-case/worst-case sensitivity analysis, in which all unreported cases were assumed in turn to possess and then to lack the feature; the resulting bounds are reported in Section 4 alongside the complete-case estimate. Frequency findings based on a small number of documented cases are reported as “in all documented cases” rather than as universal. Exact binomial 95% confidence intervals (Clopper–Pearson method) were calculated for key sonographic proportions; this method was preferred over the normal approximation because it remains valid for small samples and for proportions close to 0% or 100%. All analyses were descriptive. Because the pooled data derive from individual case reports rather than a sample from a defined population, no inferential testing or meta-analytic modeling was performed. In particular, differences across malignancy categories (benign, malignant, uncertain) are presented as counts and within-category proportions only, without significance testing: the subgroups are too small for hypothesis testing to be informative, and a non-significant result could not be distinguished from a true absence of association. Missing data were treated by available-case analysis (excluded from the denominator for the affected parameter only). Statistical analyses were performed in Python 3 (Python Software Foundation) with the SciPy library; the analysis script and raw data are publicly available at the DOI cited in Section 3.1 to allow full reproduction of every reported value.

## 4. Results

### 4.1. Search Results

The database search retrieved 386 records in total: PubMed/MEDLINE (n = 244), Scopus (n = 140), and the Cochrane Library (n = 2). After removal of 94 duplicate records, 292 titles and abstracts were screened; 135 were excluded at this stage (133 did not describe a case of uterine PEComa and two were treatment-only studies without ultrasound data). Of the 157 reports sought for full-text retrieval, 28 were not obtainable. Among the 129 reports assessed for eligibility, 111 were excluded due to absence of a detailed ultrasound description (n = 105), review article without an original case (n = 3), and non-English full text (n = 3). Eighteen studies were ultimately included, providing data for 30 patients with documented sonographic findings; together with our institutional index case (Section 2), this yielded a pooled analytic dataset of 31 cases. The PRISMA 2020 [10] flow diagram is presented in Figure 4, and the PRISMA 2020 checklist is provided as Supplementary Material (Table S1). The index case described in Section 2 was not included in the 30 literature-derived cases.

### 4.2. Study Characteristics and Quality Assessment

The 18 included studies comprised 13 case reports, four case series, and one letter to the editor with case description, published between 2004 and 2024. The methodological characteristics of each study and the corresponding JBI scores are summarized in Table 1 and Table 2. JBI scores ranged from 7/8 to 8/8 across all included studies, reflecting overall high methodological quality of reporting. The single most common deficiency was non-reporting of adverse events (Q7), scored as No or Unclear in 11 of 18 studies (61.1%), which is expected given the short follow-up of most case reports and does not reflect a substantive risk of bias in the sonographic data. The narrow range of JBI scores (7/8 to 8/8) reflects a ceiling effect inherent to the JBI Case Report checklist when applied to peer-reviewed published case reports, which by definition have undergone editorial review and tend to report core clinical information systematically.

### 4.3. Patient Characteristics

The 31 pooled patients had a median age of 41 years (IQR 28.5–52.5; mean 43.7; range 23–80; n = 22/31 with age data available). The most frequent presenting symptom was AUB (19/31, 61.3%), followed by pelvic pain (15/31, 48.4%) and asymptomatic or incidental detection (5/31, 16.1%). Lesion location was reported in 26/31 cases: submucosal in seven, subserosal in seven, cervical in five, intramural in three, broad ligament in three, and transmural in one; location was not specified in 5/31 cases. Demographic and clinical characteristics are summarized in Table 3.

### 4.4. Sonographic Features

Maximum lesion diameter ranged from 10 to 140 mm, with a median of 57 mm (IQR 47.25–78.25; mean 64.4 mm; n = 20/31 with size data). Among the 25 cases for which echogenicity was documented, 14 (56.0%; 95% CI: 34.9–75.6%) were heterogeneous and 11 (44.0%; 95% CI: 24.4–65.1%) were homogeneous; 6/31 cases did not report this parameter. Submucosal lesions appeared more frequently homogeneous (4/5, 80%) compared with subserosal (3/6, 50%) and intramural lesions (1/3, 33%), although the small numbers per subgroup preclude definitive conclusions about location-dependent echogenicity patterns. Acoustic shadowing was documented in 10 of 31 cases and was absent in all 10 (100%; 95% CI: 69.2–100.0%); this parameter was not reported in the remaining 21 cases. Irregular margins were documented in 7/23 cases (30.4% (95% CI: 13.2–52.9%)), while regular margins were recorded in 16/23 (69.6% (95% CI: 47.1–86.8%)); 8/31 cases did not describe margin morphology. Internal cystic areas were present in 2/16 cases with this parameter documented (12.5% (95% CI: 1.6–38.3%)), all in lesions subsequently classified as malignant or uncertain. Individual sonographic characteristics for all 31 cases are detailed in Table 4. Given the high proportion of unreported parameters, a best-case/worst-case sensitivity analysis was performed for the two estimates most affected by missing data. For absence of acoustic shadowing (documented in 10/31 cases), the complete-case estimate of 100% (10/10) is bounded between 100% (if all 21 unreported cases also lacked shadowing) and 32.3% (10/31; 95% CI 16.7–51.4%) in the extreme scenario in which every unreported case had shadowing present. For moderate-to-abundant vascularisation (Color Score 3–4, documented in 12/31 cases), the complete-case estimate of 91.7% (11/12) is bounded between 96.8% (30/31) and 35.5% (11/31; 95% CI 19.2–54.6%) under the analogous extreme assumptions. While the central estimates are high, they are sensitive to the unreported cases and should be read as describing the documented cases rather than established population frequencies.

### 4.5. Vascularisation

Doppler vascular data were available in 23 of 31 cases, of which 12 included a formal Color Score (CS). Of these 12, 11 (91.7%; 95% CI: 61.5–99.8%) exhibited CS 3 or CS 4 (moderate-to-abundant vascularisation); the single remaining case was reported as CS 2 (8.3%). Applying a maximum-score rule, one case documented as CS 2–3 was counted within the CS 3–4 group. Qualitative vascular descriptions were available in an additional 11 cases; the pattern was most commonly characterized as a diffuse or centrifugal network with tortuous vessels radiating from the center to the periphery, or as a peripheral ring-like vascular pattern with intratumoral feeding vessels. No case reported an absence of flow in all the visible tumor tissue, with the exception of central avascular necrotic foci in complex masses.

Standardized ultrasound terminology was explicitly applied in four of 18 studies (22.2%): Wang et al. [20] (MUSA and IETA applied to all six cases in their series), Fontana et al. [21] (MUSA), Dashraath et al. [16] (MUSA), and Mor et al. [15] (IETA). The remaining 14 studies (77.8%) used non-standardized descriptive terminology, limiting cross-study comparability of vascular and echotextural characterization.

### 4.6. Preoperative Diagnosis

The preoperative diagnosis was documented in 29 of 31 cases. Of these, none was correctly identified as PEComa prior to surgery, yielding a preoperative misdiagnosis rate of 100% (95% CI: 88.1–100.0%) (29/29). The most frequent misdiagnosis was uterine leiomyoma or fibroid (12/29, 41.4%; 95% CI 23.5–61.1%). Other misdiagnoses were uterine sarcoma (6/29, 20.7%; 95% CI 8.0–39.7%), cervical carcinoma (3/29, 10.3%; 95% CI 2.2–27.4%), and a further group of diagnoses (8/29, 27.6%; 95% CI 12.7–47.2%) that included retained products of conception, a smooth-muscle tumor of uncertain malignant potential, hydatidiform mole, endometrial polyp, unspecified mesenchymal uterine neoplasm, an indeterminate mass, and ovarian cyst.

### 4.7. MRI Correlation

MRI was performed in 12 of 31 patients (38.7%). Among the eight cases in which both ultrasonographic and MRI findings were described with sufficient detail for direct comparison (Table 5), concordance between the two modalities was consistently high. On MRI, PEComas typically appeared as T2-hyperintense masses with avid and rapid post-contrast enhancement, directly mirroring the high CS observed on Color Doppler. Haemorrhagic foci appeared as T1-hyperintense areas in complex cases, and non-enhancing necrotic regions corresponded to the cystic-hypoechoic areas detected on ultrasound. Large-caliber vessel flow voids on MRI corresponded to the prominent feeding vessels identified on Doppler in the most vascular cases. Concordance between US and MRI was documented qualitatively in all 8 described cases; the four remaining MRI-examined cases did not include a direct US-MRI comparison in the original reports.

## 5. Discussion

### 5.1. Sonographic Profile of Uterine PEComa

Our review found a consistent sonographic profile. Acoustic shadowing was absent in all documented cases (10/10, 100%; 95% CI 69.2–100.0%). Vascularisation on Color Doppler was high (Color Score 3–4 in 11/12, 91.7%; 95% CI 61.5–99.8%). The lesions were mostly solid, with variable echogenicity (heterogeneous in 56.0%; 95% CI 34.9–75.6%). Every case was misdiagnosed before surgery (100%, 29/29). Cystic areas and irregular margins were more common in cases later classified as malignant. Our index case matched this profile. It was a solid, heterogeneous lesion of 58 mm with high vascularisation (Color Score 3), absent acoustic shadowing, and irregular margins, and it was malignant on histology. Its inclusion in the pooled data is therefore justified, and its irregular margins agree with the pattern seen in the malignant cases.

### 5.2. Differential Diagnosis

Uterine PEComa is hard to diagnose because it looks like more common uterine lesions. The most frequent misdiagnosis in the reviewed cases was leiomyoma. This matters for management. Leiomyomas are often managed conservatively, so mistaking a malignant mesenchymal tumor for a leiomyoma can lead to inadequate treatment.

Leiomyosarcoma was the second most common misdiagnosis (20.7% of cases). Like PEComa, it appears as a hypervascular irregular mass and may contain central necrosis. Irregular margins are more common in leiomyosarcoma than in PEComa. The two often cannot be separated without histology. A rapidly growing myometrial mass with these features should raise suspicion of a malignant mesenchymal tumor and prompt referral.

**Figure 5:**
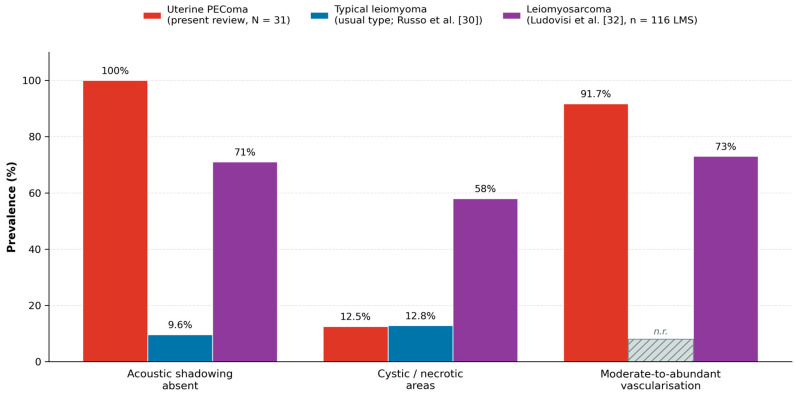
Comparative prevalence of three sonographic features in uterine PEComa, typical leiomyoma and leiomyosarcoma. Each value is the exact figure reported by the cited source: PEComa data are from the present pooled series (N = 31); typical leiomyoma data are from Russo et al. [30] (usual-type subset); leiomyosarcoma data are from Ludovisi et al. [32] (n = 116 leiomyosarcomas). The combination of absent acoustic shadowing in every documented PEComa case and moderate-to-abundant vascularisation in over 90% separates PEComa from typical leiomyoma; overlap with leiomyosarcoma remains substantial. The vascularisation value for typical leiomyoma is not reported (n.r.) as a single systematic percentage in the cited source.

Table 6 summarizes the key sonographic features that may aid in the differential diagnosis of uterine PEComa against typical leiomyoma, leiomyosarcoma, and smooth-muscle tumor of uncertain malignant potential (STUMP), drawing on the present pooled data and on the comparative ultrasound literature [29,30,31,32]. These comparative patterns are visualized in Figure 5.

Three recent MUSA-based studies offer useful comparison. De Bruyn et al. [29] compared 16 uterine sarcomas with 91 leiomyomas using independent dual-observer MUSA, and identified irregular tumor borders, non-uniform echogenicity, moderate-to-abundant intralesional vascularity, the presence of cystic areas and the absence of calcifications as the features most suggestive of malignancy; interobserver agreement for most MUSA terms was moderate. Russo et al. [30], characterizing usual-type leiomyomas and their variants, reported that acoustic shadowing was very common in usual-type leiomyomas (90.4%) and slightly less frequent in variants (79.1%), whereas cystic areas were more prevalent in variants (33.2%) than in usual-type leiomyomas (12.8%), and variant leiomyomas were larger (median 82.5 vs. 70 mm). These data indicate that, even among benign leiomyomas, the variant forms can display features overlapping with those of PEComa, such as reduced shadowing and cystic change. Nguyen et al. [31], in a series of 78 uterine sarcomas from Vietnam, confirmed that irregular borders (48.7%), moderate-to-rich vascularisation (57.7%), and cystic areas (42.3%) were common sarcoma features, while acoustic shadowing (34.6%) and calcifications (6.4%) were less frequent. Consistently, in one of the largest prospective series of uterine sarcomas, Ludovisi et al. [32] described leiomyosarcomas as large, solid, inhomogeneous masses (median diameter approximately 90 mm) with frequent cystic or necrotic areas and an absence of acoustic shadowing. These features overlap with the malignant PEComas in our series. Absent shadowing with high vascularity is therefore shared across malignant uterine mesenchymal tumors.

Within this group of lesions, uterine PEComa has a recognizable sonographic pattern. Acoustic shadowing was absent in all documented cases, vascularisation was high (CS 3–4 in 91.7%), and the margins were usually regular (69.6%). This helps separate it from typical leiomyomas, which usually show edge or fan-shaped shadowing, and from leiomyosarcomas, which more often have irregular margins. Wang et al. [20] described dispersed intratumoral vessels with a peripheral ring-like distribution, a pattern that separates PEComa from the circumferential “rim sign” usually seen in leiomyomas. The overlap with leiomyosarcoma remains substantial when vascularisation is high and cystic degeneration is present, so histology is still needed for diagnosis.

### 5.3. Comparison with Previous Literature

The present systematic review extends and complements the work of Wang et al. [20], which remains the only prior study to apply MUSA and IETA terminology to uterine PEComa. Wang et al. studied six cases and reviewed 18 more (search to September 2023). In their series most lesions were round or ovoid, uniformly hypoechoic, without shadowing, and richly vascularised (CS 3–4 in 83.3%). They proposed dispersed intratumoral vessels with a peripheral circular distribution as a diagnostic clue [20].

Our findings are broadly concordant but reveal important differences attributable to the larger and more heterogeneous pooled sample (31 cases, search up to March 2026). While the absence of acoustic shadowing and high vascularisation were confirmed as near-universal features, our review found a more balanced distribution of echogenicity (heterogeneous 56.0% vs. homogeneous 44.0%), in contrast to the predominantly uniform pattern reported by Wang et al. (66.7% uniform). This discrepancy likely reflects the inclusion of studies predating MUSA standardization, in which descriptive terminology was inconsistent.

Our review also found more irregular margins (27.3%) and confirmed that cystic areas were more common in malignant cases (Table 7). Our pooled qualitative data are consistent with the peripheral ring-like vascular pattern proposed by Wang et al., although the heterogeneity of the Doppler descriptions prevented formal quantification.

Our search was also broader. We searched three databases with no date limit up to March 2026, whereas Wang et al. searched only PubMed up to September 2023. This let us include both older reports (Fadare et al., 2004 [25]) and newer ones (Ismerat et al., 2025 [23]). We also cross-tabulated the sonographic features by malignancy category (Table 7).

### 5.4. Histopathological Correlates and Prognostic Stratification

Accurate prognostic stratification also matters. Garzon et al. [33], who demonstrated that the modified Folpe classification outperforms the original Folpe criteria in predicting recurrence and disease-specific death in a pooled cohort of 85 uterine PEComas, demonstrated that these findings suggest that the Folpe criteria applied in the present review, while widely used, may underestimate the malignant potential of borderline cases. Future studies integrating sonographic risk features with refined histopathological classification systems may improve preoperative risk stratification.

Only four of 18 included studies (22.2%) applied standardized ultrasound terminology (MUSA or IETA). This low adoption limits the literature on rare uterine tumors, because without a shared vocabulary data cannot be pooled or compared reliably across reports. The MUSA consensus group [8] provides precise, reproducible definitions for uterine mass echogenicity, margins, acoustic shadow, vascular pattern, and Color Score that are directly applicable to PEComa characterization. The IETA framework [9] similarly standardizes endometrial and submucosal lesion description. Adoption of these frameworks in future PEComa case reports would substantially improve the quality and comparability of the evidence base.

Among the 17 cases (54.8%) with histopathological malignancy data available, sonographic features were summarized descriptively by malignancy category (Table 7).

Irregular margins were observed in 4/8 malignant cases (50.0%) compared with 0/4 benign cases (0%). Cystic areas were present in 2/5 malignant cases (40.0%) but in none of the 5 benign cases with this parameter documented (0%). All malignant cases with Color Score data exhibited CS 3–4 (5/5, 100%), compared with 3/4 benign cases (75%). Acoustic shadowing was absent in every documented case regardless of malignancy category. Median lesion diameter was numerically larger in malignant cases (58 mm; range 39–105; n = 7) than in benign cases (44.5 mm; range 34–55; n = 2). The two uncertain malignant potential cases both showed heterogeneous echogenicity (2/2, 100%), compared with 3/6 in malignant and 3/6 in benign cases, indicating that the echogenicity pattern alone cannot reliably discriminate malignancy status. These comparisons are descriptive only and were not tested for significance, given the small subgroups.

Although no formal significance testing was performed, the observed trends, particularly the enrichment of cystic areas (40.0% vs. 0%) and irregular margins (50.0% vs. 0%) in malignant compared with benign cases, are consistent with the broader uterine sarcoma literature [29,30] and warrant confirmation in larger series. A sensitivity analysis was performed by reclassifying the two uncertain malignant potential cases (Verbeeck et al. [14] and Fontana et al. [19]) into the malignant category. Under this alternative grouping (malignant n = 11, benign n = 6), irregular margins remained more frequent in malignant cases (5/10, 50.0%) than in benign cases (0/4, 0%), and cystic areas were present in 2/7 malignant cases (28.6%) versus 0/5 benign cases (0%). The direction of all feature distributions was qualitatively unchanged compared with the primary analysis, confirming that the descriptive trends are robust to the classification of borderline cases.

### 5.5. Implications for Clinical Practice

For confirmed or suspected malignant PEComa, total hysterectomy with BSO remains the cornerstone of treatment [34].

In advanced or metastatic disease, mTOR-pathway inhibition (e.g., nab-sirolimus, the only agent with a positive phase II trial) is the principal targeted systemic option. A detailed treatment discussion is beyond the imaging scope of this review; the key clinical point is that earlier sonographic recognition of PEComa would enable timely, appropriately planned surgery and, where needed, referral for systemic therapy [4].

The role of conventional chemotherapy and radiotherapy remains undefined, with no prospective evidence supporting their routine use [35]. Centralisation of care in referral centers with sarcoma expertise is therefore recommended [4,35].

These treatment considerations fall outside the imaging scope of this review, but they show why early sonographic suspicion matters: recognizing PEComa before surgery can lead to timely referral for appropriate treatment.

### 5.6. Limitations

Several important limitations of this systematic review must be explicitly acknowledged. First, the entire evidence base consists of case reports and small case series (Level IV evidence), precluding meta-analysis and limiting the strength of any derived conclusions. The absence of prospective cohort studies or case–control designs comparing PEComa with other myometrial lesions means that the sonographic profile identified here cannot be validated for diagnostic accuracy (sensitivity, specificity, positive predictive value).

Second, a substantial proportion of sonographic parameters were not reported across included cases: acoustic shadowing was documented in only 10/31 cases (32%), Color Score in 12/31 (39%), cystic areas in 16/31 (52%), and histopathological malignancy classification in 17/31 (55%). This high rate of missing data directly limits the reliability and generalisability of the corresponding frequency estimates and of the descriptive subgroup comparison presented in Table 7, which was therefore not subjected to significance testing (see Section 3.7). The pattern of missing data is likely non-random (informative missingness), as earlier publications and studies not applying MUSA/IETA terminology tended to report fewer sonographic parameters, potentially biasing frequency estimates toward features that are more conspicuous or more frequently assessed.

Third, ultrasound examinations in the included studies were performed by different operators with different equipment, without standardized protocols, and without blinding to clinical information; operator-dependent variability and machine-specific differences in sensitivity (e.g., low-frequency vs. high-frequency probes, Color vs. Power Doppler) cannot be quantified. The fact that only 4/18 studies (22.2%) applied MUSA or IETA standardized terminology compounds this limitation, as non-standardized descriptions may have introduced systematic classification bias.

Fourth, the review protocol was not prospectively registered in PROSPERO or any other protocol registry, which may introduce concerns regarding potential selective reporting. However, the protocol was developed a priori and is publicly available at the DOI cited in Section 3.1. Fifth, the search was restricted to three databases (PubMed/MEDLINE, Scopus and the Cochrane Library) and to English-language, full-text publications; Embase, Web of Science, IEEE Xplore and gray literature were not searched, and non-English reports were excluded at the eligibility stage. Because several uterine PEComa series originate from non-English-speaking regions, particularly East Asia, this may have introduced language and selection bias and may have led to the omission of eligible cases, potentially limiting the completeness and generalisability of the pooled estimates.

Sixth, publication bias is a recognized concern in systematic reviews of rare tumors based exclusively on case reports: cases with atypical or dramatic presentations may be preferentially published, while cases with unremarkable sonographic features may go unreported. The extent to which the sonographic profile described in this review reflects the true spectrum of uterine PEComa, as opposed to a publishing-favored subset, cannot be determined. Finally, a recent case by Nejković et al. [36] described a uterine PEComa presenting exclusively as non-specific endometrial thickening without a distinct sonographic mass, highlighting that the sonographic profile identified in this review applies specifically to mass-forming PEComas and may not capture the full clinical spectrum of disease presentation.

## 6. Conclusions

This systematic review of 31 cases of uterine PEComa with documented sonographic data identifies a reproducible sonographic profile. Acoustic shadowing was absent in all documented cases (100%; 95% CI 69.2–100.0%). Vascularisation was moderate to abundant (CS 3–4 in 91.7%; 95% CI 61.5–99.8%). Echogenicity was variable but mostly solid (heterogeneous 56.0%; 95% CI 34.9–75.6%; homogeneous 44.0%), and margins were usually regular (69.6%; 95% CI 47.1–86.8%). Irregular margins and cystic areas were more common in malignant cases. The combination of absent shadowing and high vascularisation separates PEComa from typical leiomyomas and should prompt further investigation. Every case in this review was misdiagnosed before surgery, which shows how difficult the diagnosis is. In practice, a myometrial mass should raise suspicion of PEComa when it is solid, without flow (CS ≥ 3), and dispersed intratumoral vessels with a peripheral ring-like distribution. When this profile is encountered, referral for MRI and management in a center with sarcoma expertise is advised. The low adoption of MUSA/IETA standardized terminology in published PEComa reports (22.2% of studies) represents a significant barrier to evidence synthesis. Systematic application of these frameworks in future case reports is strongly advocated to enable meaningful cross-study comparison and to progress toward evidence-based sonographic criteria for this rare entity.

## Figures and Tables

**Table 1:** Methodological characteristics and total JBI Critical Appraisal score of the included studies (N = 18). The per-item breakdown of the JBI score is given in Table 2.

Author (Year) [Ref]	Study Design	n	Year	JBI Score
Tilstra et al. (2015) [11]	Case Report	1	2015	8/8
Rodriguez et al. (2024) [12]	Case Report	1	2024	8/8
Tirumani et al. (2014) [13]	Case Series	3	2014	8/8
Verbeeck et al. (2016) [14]	Case Report	1	2016	8/8
Mor et al. (2021) [15]	Letter/Case Report	1	2021	8/8
Dashraath et al. (2022) [16]	Case Report	1	2022	7/8
Kollabathula et al. (2021) [17]	Case Report	1	2021	8/8
Xu et al. (2024) [18]	Case Report	1	2024	7/8
Guo et al. (2019) [19]	Case Report + Review	1	2019	7/8
Wang et al. (2024) [20]	Case Series + Review	6	2024	7/8
Fontana et al. (2020) [21]	Case Report + Review	1	2020	8/8
Xholli et al. (2021) [22]	Case Report	1	2021	8/8
Ismerat et al. (2025) [23]	Case Report	1	2025	7/8
Socolov et al. (2016) [24]	Case Report + Review	1	2016	7/8
Fadare et al. (2004) [25]	Case Report + Review	1	2004	7/8
Papoutsis et al. (2019) [26]	Case Report	1	2019	7/8
Liu et al. (2019) [27]	Case Series	2	2019	7/8
Shan et al. (2019) [28]	Case Series + Review	5	2019	7/8

JBI, Joanna Briggs Institute. n, number of cases with detailed ultrasonographic data per study.

**Table 2:** Item-level JBI Critical Appraisal Checklist scores (Q1–Q8) for the included studies (N = 18). Total scores and study characteristics are reported in Table 1.

Author (Year)	Q1	Q2	Q3	Q4	Q5	Q6	Q7	Q8	Total
Tilstra et al. (2015) [11]	Y	Y	Y	Y	Y	Y	Y	Y	8/8
Rodriguez et al. (2024) [12]	Y	Y	Y	Y	Y	Y	Y	Y	8/8
Tirumani et al. (2014) [13]	Y	Y	Y	Y	Y	Y	Y	Y	8/8
Verbeeck et al. (2016) [14]	Y	Y	Y	Y	Y	Y	Y	Y	8/8
Mor et al. (2021) [15]	Y	Y	Y	Y	Y	Y	Y	Y	8/8
Dashraath et al. (2022) [16]	Y	Y	Y	Y	Y	Y	N	Y	7/8
Kollabathula et al. (2021) [17]	Y	Y	Y	Y	Y	Y	Y	Y	8/8
Xu et al. (2024) [18]	Y	Y	Y	Y	Y	Y	N	Y	7/8
Guo et al. (2019) [19]	Y	Y	Y	Y	Y	Y	N	Y	7/8
Wang et al. (2024) [20]	Y	Y	Y	Y	Y	Y	N	Y	7/8
Fontana et al. (2020) [21]	Y	Y	Y	Y	Y	Y	Y	Y	8/8
Xholli et al. (2021) [22]	Y	Y	Y	Y	Y	Y	Y	Y	8/8
Ismerat et al. (2025) [23]	Y	Y	Y	Y	Y	Y	N	Y	7/8
Socolov et al. (2016) [24]	Y	Y	Y	Y	Y	Y	N	Y	7/8
Fadare et al. (2004) [25]	Y	Y	Y	Y	Y	Y	N	Y	7/8
Papoutsis et al. (2019) [26]	Y	Y	Y	Y	Y	Y	N	Y	7/8
Liu et al. (2019) [27]	Y	Y	Y	Y	Y	Y	N	Y	7/8
Shan et al. (2019) [28]	Y	Y	Y	Y	Y	Y	N	Y	**7/8**

Q1: Demographic characteristics; Q2: Patient history; Q3: Current clinical condition; Q4: Diagnostic tests and results; Q5: Intervention/treatment and outcomes; Q6: Post-intervention condition; Q7: Adverse events; Q8: Take-away lessons. Y = Yes; N = No or Unclear.

**Table 3:** Clinical and demographic characteristics of the study population (N = 31).

Characteristic	Result
Age (years), median (IQR; mean, range)	Median 41 (IQR 28.5–52.5; mean 43.7, range 23–80); n = 22/31 with available data
Clinical Presentation, n/31 (%)	
Abnormal Uterine Bleeding (AUB)	19 (61.3%)
Pelvic Pain	15 (48.4%)
Asymptomatic/Incidental Finding	5 (16.1%)
Two or more symptoms	11 (35.5%)
Lesion Location, n/31 (%)	
Submucosal	7 (22.6%)
Subserosal	7 (22.6%)
Cervical	5 (16.1%)
Intramural	3 (9.7%)
Broad Ligament	3 (9.7%)
Transmural	1 (3.2%)
Not Reported	5 (16.1%)
Maximum Lesion Diameter (mm), median (IQR; mean, range)	Median 57 (IQR 47.25–78.25; mean 64.4, range 10–140); n = 20/31 with available data
Preoperative Misdiagnosis, n/29 (%) †	29/29 (100%)
Uterine Leiomyoma or Fibroid	12/29 (41.4%)
Uterine Sarcoma	6/29 (20.7%)
Cervical Carcinoma	3/29 (10.3%)
Other *	8/29 (27.6%)

* Other diagnoses included: retained products of conception, a smooth-muscle tumor of uncertain malignant potential, hydatidiform mole, endometrial polyp, unspecified mesenchymal uterine neoplasm, ovarian cyst, and an indeterminate mass. † Preoperative diagnosis was not documented in 2 of 31 cases. Abbreviations: AUB, Abnormal uterine bleeding; n, number of cases in category.

**Table 4:** Individual sonographic and Doppler characteristics of all included cases (N = 31).

Author	Case	Echogenicity	Vascular Pattern	CS	Shadowing	Cystic	Margins	Diam. (mm)	MUSA/IETA
Tilstra et al. [11]	—	Heterogeneous	No central flow	NR	NR	Absent	NR	55	No
Rodriguez et al. [12]	1	Heterogeneous	Rich neovascular network; coiling pedicle	CS 4	Absent	Absent	Regular	56	No
Tirumani et al. [13]	1	Heterogeneous	Non-significant	NR	NR	Absent	Regular	NR	No
Tirumani et al. [13]	2	Heterogeneous	Non-significant	NR	NR	Absent	Regular	—	No
Tirumani et al. [13]	3	Heterogeneous	Non-significant	NR	NR	Absent	Irregular	—	No
Verbeeck et al. [14]	1	Heterogeneous	Rich vascular network	NR	NR	NR	Regular	100	No
Mor et al. [15]	1	Homogeneous	Irregular vascularity	CS 2–3	NR	Absent	Regular	28	IETA
Dashraath et al. [16]	1	Homogeneous	Numerous branching feeding vessels	CS 4	Absent	Present	Regular	59	MUSA
Kollabathula et al. [17]	1	NR	NR	NR	NR	NR	Regular	105	No
Xu et al. [18]	1	NR	NR	NR	NR	NR	NR	51	No
Guo et al. [19]	1	Heterogeneous	NR	NR	NR	NR	Regular	—	No
Wang et al. [20]	1	Heterogeneous	Dispersed intratumoral + peripheral ring-like	CS 3	Absent	Absent	Regular	—	MUSA/IETA
Wang et al. [20]	2	Heterogeneous	Dispersed intratumoral + peripheral ring-like	CS 4	Absent	Absent	Regular	—	—
Wang et al. [20]	3	Homogeneous	Dispersed intratumoral + peripheral ring-like	CS 4	Absent	Absent	Regular	—	—
Wang et al. [20]	4	Homogeneous	Abundant	CS 2	Absent	Absent	Regular	—	—
Wang et al. [20]	5	Homogeneous	Abundant	CS 3	Absent	Absent	Irregular	—	—
Wang et al. [20]	6	Homogeneous	Abundant	CS 4	Absent	Present	Irregular	—	—
Fontana et al. [21]	1	Heterogeneous	Abundant	CS 4	Absent	Absent	Regular	39	MUSA
Xholli et al. [22]	1	Heterogeneous	Rich vascularity	CS 4	NR	Absent	Irregular	71	No
Ismerat et al. [23]	1	NR	NR	NR	NR	NR	Irregular	50	No
Socolov et al. [24]	1	Heterogeneous	Extremely rich central network	NR	NR	NR	Irregular	66	No
Fadare et al. [25]	1	NR	NR	NR	NR	NR	Regular	10	No
Papoutsis et al. [26]	1	Homogeneous	Moderate	NR	NR	NR	NR	34	No
Liu et al. [27]	1	NR	NR	NR	NR	NR	Regular	100	No
Liu et al. [27]	2	NR	NR	NR	NR	NR	Regular	140	No
Shan et al. [28]	1	Heterogeneous	Rich	NR	NR	NR	NR	108	No
Shan et al. [28]	2	Homogeneous	NR	NR	NR	NR	NR	35	No
Shan et al. [28]	3	Homogeneous	Abundant	NR	NR	NR	NR	52	No
Shan et al. [28]	4	Homogeneous	Abundant	NR	NR	NR	NR	71	No
Shan et al. [28]	5	Homogeneous	NR	NR	NR	NR	NR	NR	No
Present case report (Section 2.)	1	Heterogeneous	Central and peripheral	3	Absent	No	Irregular	58	Yes

CS, Color Score; MUSA, Morphological Uterus Sonographic Assessment; IETA, International Endometrial Tumor Analysis. NR means the parameter was not reported in the source study. The symbol “—” means the parameter does not apply to that case. Percentages in the Results are based only on cases in which the parameter was reported, so NR cases are excluded from the denominator.

**Table 5:** Correlation between ultrasound and MRI findings in uterine PEComa: cases with both modalities documented (n = 8).

Author (Year)	Ultrasound Findings	MRI T1/T2 Signal	MRI Enhancement	Key Concordance Point
Tilstra et al. (2015) [11]	Solid, heterogeneous, no cystic area	T1: isointense; T2: hyperintense	Intense, rapid enhancement	High vascularity confirmed on both modalities
Verbeeck et al. (2016) [14]	Solid, peripheral and internal flow (CS 4)	T1: isointense; T2: markedly hyperintense	Strong early enhancement	T2 hyperintensity correlates with high cellularity/vascularity
Tirumani et al. (2014) [13]	Large mass, irregular margins, hypervascular	T1: heterogeneous; T2: heterogeneous	Prominent flow voids (large vessels)	Large-caliber vessels on Doppler correspond to MRI flow voids
Rodriguez et al. (2024) [12]	Solid, CS 3, no acoustic shadowing	T1: low signal; T2: high signal	Diffuse, heterogeneous enhancement	Solid consistency and absence of calcifications confirmed bimodally
Dashraath et al. (2022) [16]	Complex cystic-solid mass, CS 4	T1: hyperintense foci (hemorrhage); T2: high signal	Rapid enhancement of solid components	Necrotic/haemorrhagic changes identified on both modalities
Mor et al. (2021) [15]	Homogeneous, solid, CS 2–3	T1: isointense; T2: slightly hyperintense	Homogeneous enhancement	Regular margins and solid consistency concordant
Ismerat et al. (2025) [23]	Solid-cystic, rich internal flow	T1: heterogeneous; T2: hyperintense	Intense enhancement with non-enhancing necrotic areas	US cystic areas correspond to MRI necrosis
Kollabathula et al. (2021) [17]	Solid, circumscribed, moderate flow	T1: iso-to-low; T2: hyperintense	Vivid enhancement	Well-defined margins concordant on both modalities

CS, Color Score; MRI, Magnetic Resonance Imaging; T1, T1-weighted sequence; T2, T2-weighted sequence.

**Table 6:** Comparative sonographic features of uterine PEComa and its principal differential diagnoses (typ-ical leiomyoma, leiomyosarcoma, and STUMP). PEComa data are from the present pooled series; comparator values are reported as exact percentages or medians extracted from Russo et al. [30] (typical leiomyoma), Ludovisi et al. [32] (leiomyosarcoma) and Nguyen et al. [31] (uterine sar-coma series); for STUMP, no dedicated systematic sonographic series is available. Numerical val-ues are exact percentages or medians extracted from the cited comparator studies: Russo et al. [30] (pooled cohort of usual-type and variant leiomyomas); Ludovisi et al. [32] (n = 195 uterine sarco-mas including 116 leiomyosarcomas; values reported for the leiomyosarcoma subset where available); Nguyen et al. [31] (uterine sarcoma series). For STUMP, no dedicated systematic so-nographic series is available in the published literature.

Feature	Uterine PEComa (Present Review)	Typical Leiomyoma	Leiomyosarcoma	STUMP
Echogenicity	Heterogeneous in 14/25 (56.0%); homogeneous in 11/25 (44.0%)	Often homogeneous in usual type	Heterogeneous in 94% [32]	Limited published sonographic data
Margins	Regular in 16/23 (69.6%); irregular in 7/23 (30.4%)	Usually regular in usual type	Irregular in 63% [32]	Limited published sonographic data
Acoustic shadowing	Absent in all 10 documented cases (100%)	Present in 90.4% of usual type and 79.1% of variants [30]	Present in 29% of leiomyosarcomas (no shadowing in 71%) [32]; 34.6% [31]	Limited published sonographic data
Cystic/necrotic areas	Present in 2/16 (12.5%)	12.8% in usual type and 33.2% in variants [30]	Present in 58% of leiomyosarcomas [32]	Limited published sonographic data
Vascularity (Color Score)	CS 3–4 in 11/12 (91.7%)	Limited published sonographic data; often low Color Score in usual type	Limited published sonographic data in 73% of leiomyosarcomas [32]	Moderate-to-abundant
Maximum diameter	Median 57 mm (IQR 47–78)	Median 70 mm in usual type; 82.5 mm in variants [30]	Median 91 mm (range 7–321) [32]	Variable

**Table 7:** Sonographic features stratified by histopathological malignancy classification (N = 17 cases with known malignancy status: 9 malignant, 6 benign, 2 uncertain). Values are counts and within-category percentages; differences are presented descriptively, without significance testing (see Section 3.7).

Sonographic Feature	Benign (n)	Malignant (n)	Uncertain (n)
Echogenicity	Het 3/6 (50%)Hom 3/6 (50%)	Het 3/6 (50%)Hom 3/6 (50%)	Het 2/2 (100%)
Irregular margins	0/4 (0%)	4/8 (50.0%)	1/2 (50%)
Cystic areas present	0/5 (0%)	2/5 (40.0%)	0/2 (0%)
Color Score 3–4	3/4 (75%)	5/5 (100%)	2/2 (100%)
Shadowing absent	4/4 (100%)	5/5 (100%)	2/2 (100%)
Mean diameter (mm)	44.5 (34–55) n = 2	58.0 (39–105) n = 7	63.5 (56–71) n = 2

Het, heterogeneous; Hom, homogeneous;. Values are counts (n) and within-category percentages based on cases with available data for each parameter. Differences across categories are presented descriptively only; no significance testing was performed because the subgroups are too small for hypothesis testing to be informative (see Section 3.7). In two cases (Verbeeck et al. [14] and Fontana et al. [21]), the original reports classified the tumor as “intermediate” or “uncertain malignant potential”; these were categorized accordingly.

## Data Availability

The data supporting the results of this systematic review are available within the article and its tables. The raw data, screening logs, analysis script and review protocol are publicly available at https://doi.org/10.17605/OSF.IO/EUQBF (accessed on 10 June 2026).

## Supplementary Materials

The following supporting information can be downloaded at: https://www.mdpi.com/article/10.3390/jimaging12060268/s1, Table S1: The PRISMA 2020 checklist.

## Abbreviations

The following abbreviations are used in this manuscript:

PEComa	Perivascular Epithelioid Cell Tumor
MUSA	Morphological Uterus Sonographic Assessment
IETA	International Endometrial Tumor Analysis
AUB	Abnormal Uterine Bleeding
BSO	Bilateral Salpingo-Oophorectomy
TSC	Tuberous Sclerosis Complex
mTOR	Mammalian Target of Rapamycin
HPF	High-Power Fields
CS	Color Score
JBI	Joanna Briggs Institute
US	Ultrasound
CT	Computed Tomography
MRI	Magnetic Resonance Imaging
SMA	Smooth Muscle Actin
ER	Estrogen Receptor
FIGO	International Federation of Gynecology and Obstetrics
