# Supplementary material for: Ultrasound Features of Uterine Perivascular Epithelioid Cell Tumor (PEComa): A Systematic Review
Source: J Imaging. 2026 Jun 18;12(6):268. doi: 10.3390/jimaging12060268 (PMC13300927; doi:10.3390/jimaging12060268)
Supplement: Supplementary file 1 [file jimaging-12-00268-s001.zip › jimaging-4272807-supplementary.pdf]

Supplementary Table S1. PRISMA 2020 Checklist

Section and Topic	Item #	Checklist item	Location where item is reported
**TITLE**			
Title	1	Identify the report as a systematic review.	Title page
**ABSTRACT**			
Abstract	2	See the PRISMA 2020 for Abstracts checklist.	Abstract
**INTRODUCTION**			
Rationale	3	Describe the rationale for the review in the context of existing knowledge.	Introduction (Section 1)
Objectives	4	Provide an explicit statement of the objective(s) or question(s) the review addresses.	Introduction (Section 1, final paragraph: aims i–iii)
**METHODS**			
Eligibility criteria	5	Specify the inclusion and exclusion criteria for the review and how studies were grouped for the syntheses.	Methods 3.2 (Eligibility Criteria)
Information sources	6	Specify all databases, registers, websites, organisations, reference lists and other sources searched or consulted to identify studies. Specify the date when each source was last searched or consulted.	Methods 3.3 (Information Sources and Search Strategy); last search: 1 March 2026
Search strategy	7	Present the full search strategies for all databases, registers and websites, including any filters and limits used.	Methods 3.3 (full search strings reported for PubMed/MEDLINE, Scopus, Cochrane Library); also archived in OSF deposit: https://doi.org/10.17605/OSF.IO/EUQBF
Selection process	8	Specify the methods used to decide whether a study met the inclusion criteria of the review, including how many reviewers screened each record and each report retrieved, whether they worked independently, and if applicable, details of automation tools used in the process.	Methods 3.4 (Study Selection): two independent reviewers (L.G.Z., G.M.B.); Cohen's κ = 1.00; no automation tools used
Data collection process	9	Specify the methods used to collect data from reports, including how many reviewers collected data from each report, whether they worked independently, any processes for obtaining or confirming data from study investigators, and if applicable, details of automation tools used in the process.	Methods 3.5 (Data Extraction): two independent reviewers using a predefined extraction form; no contact with original authors; no automation tools
Data items	10a	List and define all outcomes for which data were sought. Specify whether all results that were compatible with each outcome domain in each study were sought (e.g. for all measures, time points, analyses), and if not, the methods used to decide which results to collect.	Methods 3.5; outcomes: grey-scale and Doppler sonographic features (MUSA/IETA terminology), preoperative diagnosis, malignancy category. All compatible results sought.
	10b	List and define all other variables for which data were sought (e.g. participant and intervention characteristics, funding sources). Describe any assumptions made about any missing or unclear information.	Methods 3.5; other variables: age, lesion diameter, location, symptoms. Missing data coded NR; no assumptions imputed.
Study risk of bias assessment	11	Specify the methods used to assess risk of bias in the included studies, including details of the tool(s) used, how many reviewers assessed each study and whether they worked independently, and if applicable, details of automation tools used in the process.	Methods 3.6 (Quality Assessment): JBI Critical Appraisal Checklist for Case Reports applied independently by two reviewers; no automation tools.
Effect measures	12	Specify for each outcome the effect measure(s) (e.g. risk ratio, mean difference) used in the synthesis or presentation of results.	Methods 3.7: proportions (n/N, %) with exact binomial 95% confidence intervals (Clopper–Pearson); medians with IQR and range for continuous variables.
Synthesis methods	13a	Describe the processes used to decide which studies were eligible for each synthesis (e.g. tabulating the study intervention	Methods 3.7; all eligible studies (n = 18) contributed to the pooled descriptive

		characteristics and comparing against the planned groups for each synthesis (item #5)).	synthesis at the case level (N = 31, including the institutional index case).
	13b	Describe any methods required to prepare the data for presentation or synthesis, such as handling of missing summary statistics, or data conversions.	Methods 3.7; available-case analysis per parameter; no imputation; NR cases excluded from the denominator of the affected variable only.
	13c	Describe any methods used to tabulate or visually display results of individual studies and syntheses.	Results 4.2–4.7; per-case data in Table 4; summary statistics in Table 3; comparative features in Tables 6–7; visualisations in Figures 4 and 5.
	13d	Describe any methods used to synthesize results and provide a rationale for the choice(s). If meta-analysis was performed, describe the model(s), method(s) to identify the presence and extent of statistical heterogeneity, and software package(s) used.	Methods 3.7; descriptive synthesis only. No meta-analysis: the case-report nature of the included evidence does not support meta-analytic modelling. Analyses run in Python 3 with SciPy.
	13e	Describe any methods used to explore possible causes of heterogeneity among study results (e.g. subgroup analysis, meta-regression).	Methods 3.7; descriptive subgroup comparison by histopathological malignancy category (benign / malignant / uncertain), without inferential testing (small subgroups).
	13f	Describe any sensitivity analyses conducted to assess robustness of the synthesized results.	Methods 3.7; best-case/worst-case sensitivity analysis for the two parameters most affected by missing data (acoustic shadowing absence and Color Score 3–4); reported in Results 4.4.
Reporting bias assessment	14	Describe any methods used to assess risk of bias due to missing results in a synthesis (arising from reporting biases).	Not formally assessed; addressed qualitatively in Discussion 5.6 (publication bias as the sixth limitation, given that the evidence base is exclusively published case reports of a rare tumor).
Certainty assessment	15	Describe any methods used to assess certainty (or confidence) in the body of evidence for an outcome.	Not formally assessed (e.g. no GRADE). The case-report level of the included evidence is acknowledged in Discussion 5.6 as the first limitation.
**RESULTS**			
Study selection	16a	Describe the results of the search and selection process, from the number of records identified in the search to the number of studies included in the review, ideally using a flow diagram.	Results 4.1 (Search Results); Figure 4 (PRISMA 2020 flow diagram): 386 → 292 → 157 → 129 → 18 included studies.
	16b	Cite studies that might appear to meet the inclusion criteria, but which were excluded, and explain why they were excluded.	Results 4.1; Figure 4 (full-text exclusions broken down: 105 lacking detailed ultrasound description; 3 reviews without original case; 3 non-English).
Study characteristics	17	Cite each included study and present its characteristics.	Results 4.2 (Study Characteristics and Quality Assessment); Tables 1, 2 and 4; References [11–28].
Risk of bias in studies	18	Present assessments of risk of bias for each included study.	Results 4.2; Tables 1–2 (JBI scores 7–8/8 for all included studies).
Results of individual studies	19	For all outcomes, present, for each study: (a) summary statistics for each group (where appropriate) and (b) an effect estimate and its precision (e.g. confidence/credible interval), ideally using structured tables or plots.	Table 4 (per-case sonographic data for all 31 cases, including the institutional index case).
Results of syntheses	20a	For each synthesis, briefly summarise the characteristics and risk of bias among contributing studies.	Results 4.2 (study characteristics and JBI scores). All 18 included studies were case reports or small case series.

	20b	Present results of all statistical syntheses conducted. If meta-analysis was done, present for each the summary estimate and its precision (e.g. confidence/credible interval) and measures of statistical heterogeneity. If comparing groups, describe the direction of the effect.	Results 4.3–4.7; Table 3 (summary statistics with 95% CIs); Table 6 (descriptive subgroup by malignancy).
	20c	Present results of all investigations of possible causes of heterogeneity among study results.	Results 4.4 and Table 6 (descriptive subgroup comparison by malignancy category); no significance testing performed.
	20d	Present results of all sensitivity analyses conducted to assess the robustness of the synthesized results.	Results 4.4 (best-case/worst-case bounds for acoustic shadowing absence and Color Score 3–4); Discussion 5.4 (sensitivity reclassification of uncertain malignant potential cases).
Reporting biases	21	Present assessments of risk of bias due to missing results (arising from reporting biases) for each synthesis assessed.	Discussion 5.6 (sixth limitation: publication bias in case-report literature on a rare tumor).
Certainty of evidence	22	Present assessments of certainty (or confidence) in the body of evidence for each outcome assessed.	Discussion 5.6 (case-report level evidence; qualitative certainty; no GRADE).
**DISCUSSION**			
Discussion	23a	Provide a general interpretation of the results in the context of other evidence.	Discussion 5.1–5.4 (Sonographic profile; Differential diagnosis; Comparison with previous literature; Histopathological correlates and prognostic stratification).
	23b	Discuss any limitations of the evidence included in the review.	Discussion 5.6 (first three limitations: case-report level evidence; missing data and informative missingness; operator-dependent variability and non-standardised terminology).
	23c	Discuss any limitations of the review processes used.	Discussion 5.6 (fourth and fifth limitations: lack of prospective PROSPERO registration; restriction to three databases and English-language publications).
	23d	Discuss implications of the results for practice, policy, and future research.	Discussion 5.5 (Implications for clinical practice); Section 6 (Conclusions).
**OTHER INFORMATION**			
Registration and protocol	24a	Provide registration information for the review, including register name and registration number, or state that the review was not registered.	Methods 3.1 (Study Design and Registration): the review was not prospectively registered in PROSPERO or any other protocol registry.
	24b	Indicate where the review protocol can be accessed, or state that a protocol was not prepared.	Methods 3.1; protocol publicly available at OSF deposit: https://doi.org/10.17605/OSF.IO/EUQBF
	24c	Describe and explain any amendments to information provided at registration or in the protocol.	Methods 3.5 / Discussion 5.6: the institutional index case was integrated into the pooled dataset following peer-review recommendation; no other amendments.
Support	25	Describe sources of financial or non-financial support for the review, and the role of the funders or sponsors in the review.	Funding statement: no external funding.
Competing interests	26	Declare any competing interests of review authors.	Conflicts of Interest statement: the authors declare no conflicts of interest.

Availability of data, code and other materials	27	Report which of the following are publicly available and where they can be found: template data collection forms; data extracted from included studies; data used for all analyses; analytic code; any other materials used in the review.	Data Availability Statement; all materials (data extraction form, raw extracted data, dual-reviewer screening logs, analysis script, review protocol) publicly available at OSF deposit: https://doi.org/10.17605/OSF.IO/EUQBF
